# Apical tubular complement activation and the loss of kidney function in proteinuric kidney diseases

**DOI:** 10.1093/ckj/sfae215

**Published:** 2024-07-10

**Authors:** Firas F Alkaff, Rosa G M Lammerts, Mohamed R Daha, Stefan P Berger, Jacob van den Born

**Affiliations:** Division of Nephrology, Department of Internal Medicine, University of Groningen, University Medical Center Groningen, Groningen, The Netherlands; Division of Pharmacology and Therapy, Department of Anatomy, Histology, and Pharmacology, Faculty of Medicine Universitas Airlangga, Surabaya, Indonesia; Transplantation Immunology, Department of Laboratory Medicine, University of Groningen, University Medical Center Groningen, Groningen, The Netherlands; Department of Nephrology, Leiden University Medical Center, Leiden, The Netherlands; Division of Nephrology, Department of Internal Medicine, University of Groningen, University Medical Center Groningen, Groningen, The Netherlands; Division of Nephrology, Department of Internal Medicine, University of Groningen, University Medical Center Groningen, Groningen, The Netherlands

**Keywords:** complement activation, disease progression, kidney diseases, kidney tubule, proteinuria

## Abstract

Many kidney diseases are associated with proteinuria. Since proteinuria is independently associated with kidney function loss, anti-proteinuric medication, often in combination with dietary salt restriction, comprises a major cornerstone in the prevention of progressive kidney failure. Nevertheless, complete remission of proteinuria is very difficult to achieve, and most patients with persistent proteinuria slowly progress toward kidney failure. It is well-recognized that proteinuria leads to kidney inflammation and fibrosis via various mechanisms. Among others, complement activation at the apical side of the proximal tubular epithelial cells is suggested to play a crucial role as a cause of progressive loss of kidney function. However, hitherto limited attention is given to the pathophysiological role of tubular complement activation relative to glomerular complement activation. This review aims to summarize the evidence for tubular epithelial complement activation in proteinuric kidney diseases in relation to loss of kidney function.

## INTRODUCTION

Proteinuria is a strong independent predictor of decreased kidney function [1–3]. Thus, when patients are found to have proteinuria, anti-proteinuric medication is given, preferably in combination with dietary salt intake restriction [4]. Drugs that intervene in the renin–angiotensin–aldosterone system (RAAS) are known for their anti-proteinuric properties [1]. Findings from clinical trials have shown that these drugs can slow kidney failure progression [5–7]. More recently, novel drug classes, such as sodium–glucose co-transporter 2 inhibitors, have also been shown to reduce proteinuria and slow down the progression toward kidney failure [8]. Nevertheless, despite such dietary and medication interventions [9, 10], proteinuria is only partially suppressed, where it remains to cause progressive kidney failure [7, 11].

It is thought that proteinuria contributes to progressive kidney failure via tubulointerstitial injury [3, 12, 13]. There are several mechanisms by which proteinuria can cause tubulointerstitial injury, e.g. by obstructing the tubular lumen with protein casts, by causing energy depletion and lysosomal rupture due to an excessive amount of protein overload that needs to be reabsorbed by the proximal tubular epithelial cells (PTECs), by triggering PTEC to release pro-inflammatory and pro-fibrotic cytokines and by causing intracellular accumulation of lipidated proteins that can trigger tubular apoptosis [2, 12, 13]. In addition, components within the proteinuric ultrafiltrate can cause damage to the tubular compartment directly. Among these components, complement factors may bind to the apical membranes of PTECs and initiate complement activation, which in turn is believed to play a crucial role in tubulointerstitial injury [12, 14].

The complement system is an important innate and acquired immune system component [15]. Activation and inhibition of the complement system are tightly regulated and dysregulation of this system plays a role in many diseases, including a number of kidney diseases [16–18]. Since the discovery of complement involvement in the pathogenesis of kidney diseases in 1952 [19], >10 000 articles have been published on this topic. So far, most articles on complement involvement in kidney diseases have focused predominantly on the glomeruli or peritubular capillaries [20, 21]. Limited attention has been given to tubular complement activation, although its involvement has been shown to be associated with tubular injury and a decrease in kidney function in both native kidney diseases and kidney transplantation [22–27]. Therefore, this review aims to summarize the findings describing tubular complement activation that takes place on the apical side of the tubules in proteinuric kidney diseases, including its eventual functional consequences.

## SYNTHESIS, ACTIVATION, REGULATION AND FUNCTIONS OF THE COMPLEMENT SYSTEM

The complement system consists of a set of plasma components that are linked and can be activated in a cascading manner [28]. The complement system has >30 components and activation fragments, comprised of pattern recognition molecules (PRMs), serine proteases and other components of the cascade, receptors and regulators [29]. The majority of the complement components are produced by hepatocytes [30]; however, other cells, including PTECs, are capable of synthesizing complement components under the driving force of cytokines [30–32].

**Figure 1:**
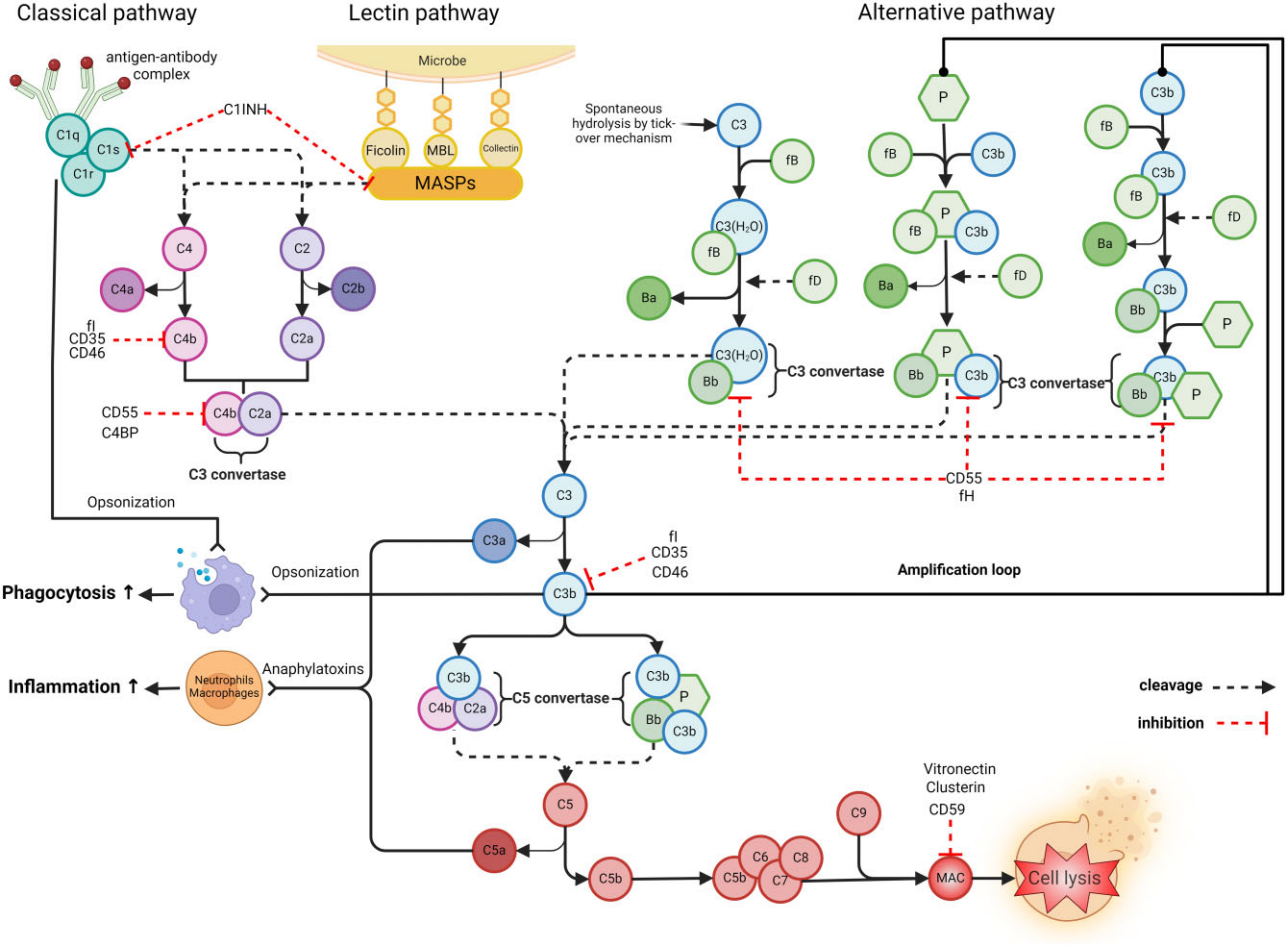
Complement activation pathways. The complement system can be initiated via three pathways: the classical pathway (triggered by immune complexes, with C1q as the PRMs) [33], the lectin pathway (initiated mainly by carbohydrates on microbial surfaces, with MBL, ficolins or collectins as the PRMs) [34] and the alternative pathway (spontaneously and constantly activated on biological surfaces via the tick-over mechanism or by properdin as the PRM) [35]. Activation of any of these pathways results in assembly of the C3 convertase that cleaves C3 into C3a and C3b, followed by assembly of the C5 convertase that cleaves C5 into C5a and C5b, and finally the formation of terminal C5b-9 membrane attack complex (MAC) [36]. The sequential and exponential cascade of complement activation subsequently triggers an inflammatory response by recruiting and activating immune cells facilitating phagocytosis and directly causing membrane cell lysis [17]. CRPs regulate complement activation and are present as membrane-bound (i.e. CD35, CD46, CD55 and CD59) proteins or in soluble form (i.e. C1 inhibitor [C1-INH], C4b-binding protein [C4BP], factor H [fH], factor I [fI], clusterin and vitronectin). The detailed function of each CRP in regulating complement activation has been described elsewhere [18].

Complement is activated by three major pathways: the classical (CP) [33], the lectin (LP) [34] and the alternative (AP) [35]. Activation of any of these pathways results in the assembly of C3 convertase, followed by the formation of C5 convertase and finally the terminal C5b-9 membrane attack complex [36]. Details on the complement system, including its functions and regulation, are illustrated in Fig. 1.

## GLOMERULAR FILTRATION BARRIER AND PROTEINURIA SELECTIVITY

In the kidney, blood is filtered by the glomerular filtration barrier (GFB) before the ultrafiltrate enters the tubular system. The GFB selectively filters proteins based on size and net charge. Under normal conditions, only plasma proteins with molecular weights <40 kDa, such as β2-microglobulin (molecular weight ≈11.8 kDa) or α1-microglobulin (molecular weight ≈31 kDa), are completely unrestricted to pass through the GFB. Meanwhile, only a fraction of proteins of intermediate molecular weight, particularly albumin (molecular weight ≈67 kDa), and none of the high molecular weight proteins such as immunoglobulin G (IgG; molecular weight ≈150 kDa), α2-macroglobulin (molecular weight ≈720 kDa) and pentameric IgM (molecular weight ≈900 kDa) can pass through the GFB. However, the small proteins and polypeptides that are able to pass through the GFB are completely reabsorbed in the proximal tubules [37, 38].

The molecular weight of plasma complement components ranges from 27 to 460 kDa [39–44]. The terminal complement complex C5b-9 has an even higher molecular weight, ≈1000 kDa [45] (Table 1). Thus, in physiological conditions, the majority of the complement factors do not pass the GFB and thus cannot result in complement activation at the apical sides of the PTECs.

**Table 1:** Complement components and their molecular weight [39–44].

Complement components	Molecular weight (in kDa)
Classical pathway	
C1q	460
C1r	90
C1s	80
Lectin pathway	
MBL	288
Ficolin-1 (M-ficolin)	384
Ficolin-2 (L-ficolin)	372
Ficolin-3 (H-ficolin)	372
Collectin-11	200–300
MASP-1	154
MASP-2	146
MASP-3	158
Classical and lectin pathways	
C2	83
C4	190
Alternative pathway	
Properdin	53
Factor B	93
Factor D	24
Common terminal pathway	
C3	185
C5	191
C6	120
C7	110
C8	151
C9	71
Complement regulators	
CD35 (CR1)	190
CD46 (MCP)	45–67
CD55 (DAF)	70
Factor H	190
Factor I	88
CD59	18

Depending on the kidney disease, the disruption of GFB leads to either selective or non-selective proteinuria. In selective proteinuria, albumin and small amounts of heavy molecular weight proteins pass through the filtration barrier and reach the tubular lumen. Due to the extra amount of protein in the pre-urine, the reabsorption process of tubular cells becomes saturated, resulting in the loss of mainly low molecular weight proteins and albumin in the urine [37, 38, 46]. In non-selective proteinuria, a large amount of high molecular weight proteins pass the GFB and be excreted in the urine [37]. Compared with selective proteinuria, non-selective proteinuria is associated with more severe tubulointerstitial injury [37, 47]. To distinguish between selective and non-selective proteinuria, selectivity indices (SIs) are used. SIs can be calculated using IgG:transferrin, IgG4:IgG1, α2-macroglobulin:albumin or IgM:albumin clearance ratios [48–50].

The presence of soluble C5b-9 (sC5b-9) in the urine originates exclusively from local tubular complement activation, with the fractional excretion of sC5b-9 being ≈100 times higher than that of IgM despite the similar molecular size [51]. This makes ultrafiltration of sC5b-9 highly unlikely, even in non-selective proteinuria. This notion is also supported by findings from various studies showing that urinary sC5b-9 levels in proteinuric patients do not correlate with sC5b-9 levels in circulation [23, 27, 52–58].

## ACTIVATION OF COMPLEMENT SYSTEM AT THE APICAL SIDE OF THE TUBULES DURING NON-SELECTIVE PROTEINURIA

**Figure 2:**
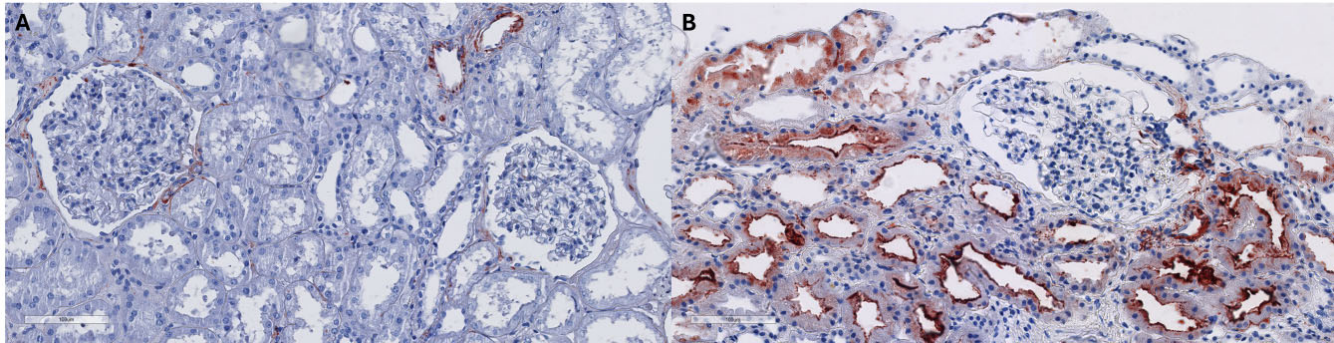
Staining of C5b-9 in a healthy and a diseased kidney. **(A)** A normal portion of a nephrectomized kidney from a patient who had a renal tumour. **(B)** A kidney biopsy of a patient with FSGS with non-selective proteinuria.

When non-selective proteinuria persists, the progression of kidney injury toward end-stage kidney failure is consistent and irreversible via proteinuria-induced tubular damage pathways [2, 12, 13]. It has been suggested that one of the main contributing factors to this tubular damage is activation of the complement system within the tubules [12, 14]. Patients with non-selective proteinuria generally have higher urinary sC5b-9 concentrations compared with patients with selective proteinuria, regardless of the primary kidney diseases [27, 51, 56], and the urinary sC5b-9 level is positively associated with the severity of tubulointerstitial injury [24, 25, 55, 59]. In addition, deposition of C5b-9 at the apical side of the tubules is only observed in patients with non-selective proteinuria, not in selective proteinuria and also not in the absence of proteinuria [52, 60] (Fig. 2). Findings from these observational studies were supported by the experimental studies using rodents, where it has been shown that activation of the complement system can mediate tubulointerstitial injury only in non-selective proteinuria, and C5b-9 deposition at the apical side of the tubules is only observed in that condition [61–64].

The susceptibility of the apical side of the tubules for complement activation during proteinuria may be related to the low expression of complement regulatory proteins (CRPs) in the tubular segment. Under healthy conditions, CD59 is the only membrane-bound CRP present at the apical side of the tubules, albeit to a limited extent compared with other segments in the kidney (i.e. glomerular and vascular segments) [65]. Thus, upon increased filtration of complement components through the defective GFB, tubular complement activation can occur more easily and may contribute to the progression of kidney injury.

## APICAL TUBULAR COMPLEMENT ACTIVATION LEADS TO TUBULOINTERSTITIAL INFLAMMATION AND FIBROSIS

Upon activation, C5b binds to the apical side of the PTECs and locally functions as a focus for the generation of C5b-9 complex. Insertion of C5b-9 into the cell membrane of PTECs results in local production of C3 [66, 67] and secretion of various pro-inflammatory cytokines such as TNF-α and IL-6 [68–70]. TNF-α and IL-6 enhance the expression of monocyte chemoattractant protein-1 (MCP-1) by the PTECs [71–73], which leads to recruitment and activation of macrophages into the interstitial area [70, 74, 75]. In addition, PTECs also recruit macrophages via extracellular vesicles (EVs) [76]. When activated, macrophages promote injury via reactive oxygen species and nitric oxide production and also by releasing pro-inflammatory cytokines [77]. Furthermore, macrophages also release pro-fibrogenic cytokines, such as transforming growth factor β (TGF-β), which in turn increases the expression of extracellular matrix components (ECMs) such as collagen type I and III and fibronectin by the interstitial myofibroblast [77, 78]. Additionally, insertion of the C5b-9 complex into the PTECs in the apical side of the tubules has also been shown to upregulate collagen type IV expression [79]. Increased deposition of ECMs is the hallmark of kidney fibrosis [80].

Along with C5b-9, C3a is also involved in the pathogenesis of tubulointerstitial injury and fibrosis. It has been reported that upon binding to its receptor at the apical side of the tubules, C3a is able to attract macrophages to infiltrate into the interstitial compartment and also stimulate PTECs to produce collagen type I and TGF-β at the basolateral side of the tubules [81–83]. Furthermore, C3a but not C5b-9 mediate the epithelial–mesenchymal transition (EMT) of the PTECs [82]. Of three major EMT signalling pathways (i.e. TGF-β/Smad, integrin/ILK and Wnt/β-catenin) [84], it has been recently demonstrated that C3a is involved in TGF-β signalling by promoting activation of the NLRP3 inflammasome [85]. Following dedifferentiation, the PTECs, through its mesenchymal properties, are able to aggravate kidney fibrosis by promoting interstitial myofibroblast differentiation and fibrogenesis by secreting cytokines and EVs [76, 86].

**Figure 3:**
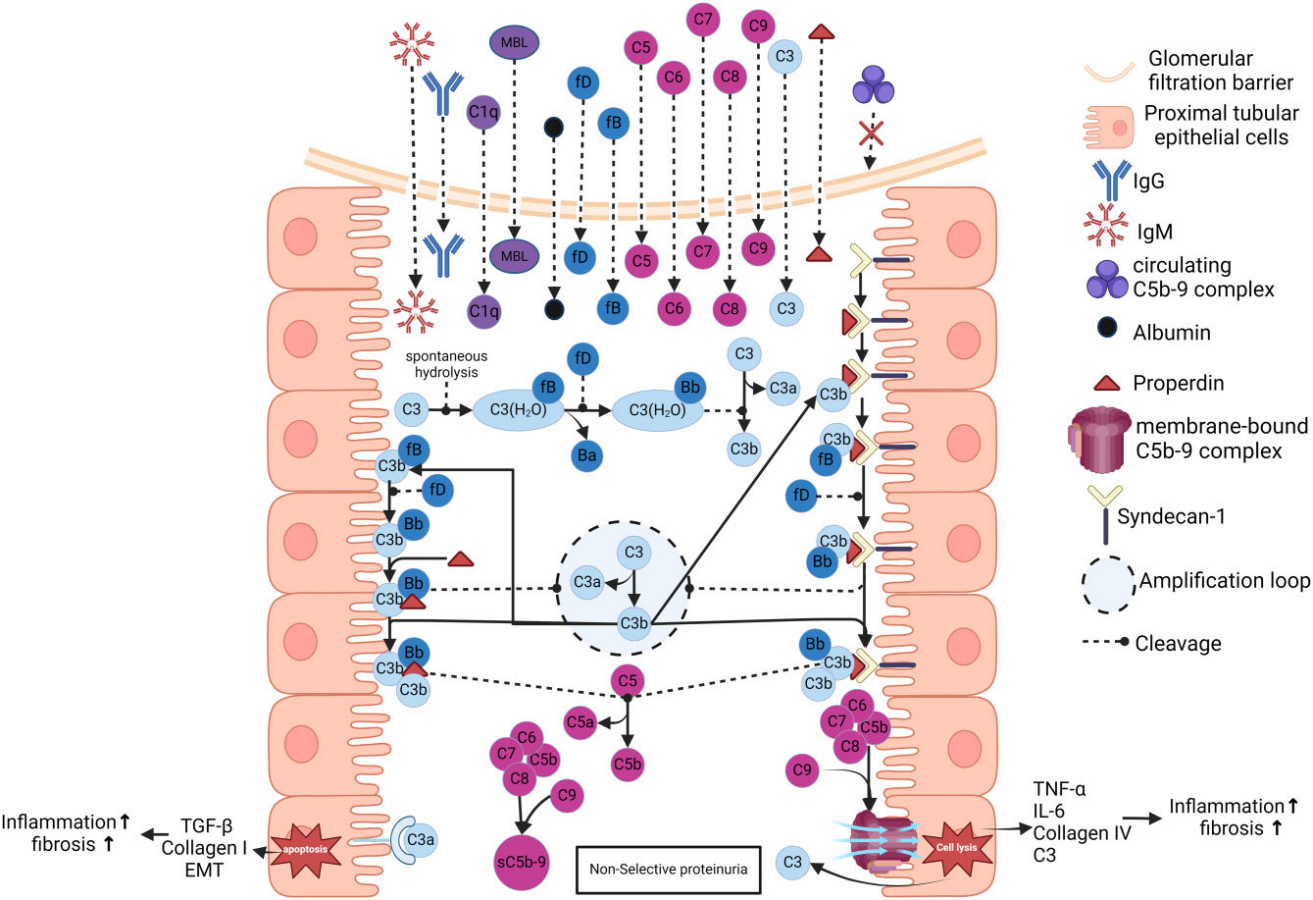
Activation pathway of the complement system at the apical side of the proximal tubules and its consequences. During non-selective proteinuria, all proteins may pass through the GFB, with the exception of sC5b-9 complex. In the tubular compartment, properdin activates the complement system upon binding to the syndecan-1 or C3b as the PRM [35, 94–96]. In contrast, pattern recognition molecules of classical and lectin pathways (represented by C1q and MBL in this illustration) do not bind to the proximal tubule epithelial cells and are directly secreted as urine. As a consequence of the complement activation, C3a binds to its receptor in the proximal tubule epithelial cells and upregulates the expression of collagen type I and TGF-β [81, 83] to the basolateral side of the proximal tubules and also induces EMT of the PTECs [82]. Next to C3a, C5b-9 induces cell lysis [69] and stimulates pro-inflammatory (TNF-α, IL-6) [68] and pro-fibrotic (collagen type IV) cytokines [79] to the basolateral side of the proximal tubules. As a result, tubulointerstitial injury (inflammation and fibrosis) occurs. See text for further details.

Next to the apical side, complement activation also takes place at the basolateral side of the tubules due to the local synthesis of complement factors, including C3, by the PTECs [31, 66, 67]. Although the basolateral side of the tubules has more CRPs than the apical side [65], excessive complement activation may eventually surpass the inhibitory capability of CRPs, leading to further tubulointerstitial injury [87]. In addition, C5a has been reported to cause DNA methylation changes by inducing aberrant epigenetic modifications. As a consequence, the Wnt4/β-catenin signalling pathway becomes more active, leading to accelerated PTEC senescence [88]. Evidently, this process aggravates tubulointerstitial fibrosis [89]. The consequences of complement activation at the apical side of the proximal tubules are illustrated in Fig. 3.

## ACTIVATION PATHWAY(S) OF THE COMPLEMENT SYSTEM AT THE APICAL SIDE OF THE PROXIMAL TUBULES

Camussi *et al*. [90] performed *in vitro* experiments using frozen kidney sections from healthy rats that were incubated with normal human serum as a source of complement. It was discovered that the AP component properdin and C3 were deposited at the apical side of the proximal tubules, whereas CP components such as C1q and C4 were absent. Later, they repeated the *in vitro* experiment using ‘healthy’ human frozen kidney sections and found similar results [91]. They hypothesized that complement activation in the proximal tubules may also occur in patients with non-selective proteinuria. They then demonstrated that C3 was deposited at the brush border of proximal tubules in patients with non-selective proteinuria, while C1q and C4 were not [92]. Later, Biancone *et al*. [69] isolated PTECs from normal human kidneys and exposed them to normal human serum. Similar to the findings from previous investigators, C1q and C4 did not bind to the PTECs, whereas properdin was found along with other downstream complement components at the surface of the PTECs.

Following the discovery that properdin can initiate the AP [93], Gaarkeuken *et al*. [94]) demonstrated that properdin can bind to the surface of PTECs and initiate AP complement activation in a dose-dependent manner. This finding has been replicated by others [22, 95]. For properdin to bind to the apical surface of PTECs, it needs heparan sulfate proteoglycans (e.g. syndecan-1) with heparan sulfate glycosaminoglycan side chains as the docking platform [95, 96]. In healthy kidneys, tubular epithelial syndecan-1/heparan sulfate is expressed on the basolateral side; however, the expression is significantly increased and translocated to an apical position in proteinuric conditions, presumably by the loss of cellular polarity [97]. The detailed activation pathway of the complement system at the apical side of the tubules is illustrated in Fig. 3.

## PRIMARY PROTEINURIC KIDNEY DISEASE WITHOUT COMPLEMENT INVOLVEMENT

Minimal change disease (MCD) is an ideal example of a kidney disease with selective proteinuria. Patients with this disease present with nephrotic syndrome with minimal or no histological change in the kidney. Hence the name minimal change is used. Due to the similarity in clinical presentations, MCD may sometimes be confused with focal segmental glomerulosclerosis (FSGS), especially if the biopsy sample is inadequate [98, 99]. However, unlike FSGS, MCD rarely progresses to kidney failure [100].

Despite the amount of proteinuria, studies have shown that patients with MCD have urinary C5b-9 levels comparable to healthy non-proteinuric control individuals [27, 51, 56, 101]. Furthermore, compared with FSGS, MCD patients have significantly lower urinary sC5b-9 levels [27, 56, 102] and a much better prognosis. Because of the selectivity of the proteinuria, urine samples of MCD patients are often used as controls alongside healthy individuals for evaluating complement activation products in other kidney diseases [27, 56, 101].

## PRIMARY PROTEINURIC KIDNEY DISEASES WITH TUBULAR COMPLEMENT INVOLVEMENT

### FSGS

FSGS is characterized by injury of podocytes that results in effacement of the podocyte foot processes [103]. In FSGS, activation of the complement system in the circulation or locally in the glomerulus does not contribute to glomerular injury [52, 57, 104, 105]. However, urinary sC5b-9 levels are significantly elevated in patients with FSGS [27, 51, 56, 57, 105, 106]. Moreover, increased urinary sC5b-9 positively correlates with the amount of proteinuria and negatively with kidney function. In addition, patients with interstitial fibrosis also show higher urinary sC5b-9 levels [57]. Next to that, FSGS patients with elevated urinary sC5b-9 have been shown to have C5b-9 deposition at the apical side of the proximal tubules [52].

Concerning the activation pathway, observational studies evaluating urinary Bb levels showed conflicting results, where some reported increased urinary Bb levels compared with the healthy controls [27, 57] and others reported that the levels were similar [105] or decreased [56]. Moreover, no study has evaluated which complement components besides C5b-9 are deposited in the tubular epithelium. Such studies would be necessary to gain better insights into how the complement activation pathway is involved on a tubular level.

In various FSGS animal models, C3 and C5b-9 have been found to be deposited at the apical side of the proximal tubules [61, 63, 70, 82, 107, 108]. Furthermore, the complement deposition correlated with the degree of tubular injury. Compared with the wild-type, tubular injury was significantly milder in C6^−/−^ rat models. In addition, interstitial fibrosis and interstitial monocyte accumulation are also milder [61, 63, 108]. In CD59^−/−^ mice, tubulointerstitial injury was more severe [107]. Similar results were reported in studies using C3^−/−^, C3aR^−/−^ and CD55^−/−^ mice [82, 107, 109]. In the adriamycin rat model, C3 deposition in the tubules did not co-localize with IgM [110]. Since co-localization of IgM with complement components indicates CP activation, this suggests that tubular C3 deposition does not come from the CP. This suggestion was strengthened by findings from another study where tubulointerstitial damage in C1q^−/^^−^ mice is similar to that in the wild-type [107]. In contrast, factor D^−/−^ mice are more protected against tubulointerstitial damage than wild-type mice [107]. Similarly, tubular complement deposition is not detected in factor B^−/−^ mice [111]. Furthermore, Zaferani *et al*. [96] also showed that properdin binds to the apical cell membranes of PTECs in the adriamycin rat model.

In summary, evidence from human and animal FSGS models showed that the complement system is activated in the tubular compartment, more specifically at the apical side of the proximal tubules. Animal models have clearly shown that AP is the principal complement activation pathway in tubular complement activation in FSGS. However, whether the same holds for humans remains to be demonstrated.

### IgA nephropathy (IgAN)

IgAN is the most common primary glomerulopathy in adults, characterized by the deposition of IgA in the mesangium of the glomerulus [112]. Systemically, the complement system is not activated in patients with IgAN, as the plasma sC5b-9 level is not increased [94]. However, complement is activated locally in the mesangium by immune complex deposition, as C5b-9 deposition was present in the mesangium of patients with IgAN [20, 113, 114]. In addition, urinary sC5b-9 was found to be significantly higher in patients with IgAN compared with healthy controls, and the level was positively correlated with proteinuria and serum creatinine. Furthermore, urinary sC5b-9 significantly correlated with proximal tubular injury markers [23, 24, 27, 55, 115]. Although, to our knowledge, no study has linked the urinary sC5b-9 level with the intensity of the apical C5b-9 deposition in the tubules, the urinary sC5b-9 level does not correlate with the circulating sC5b-9 level [54], suggesting local complement activation in the tubular compartment.

Regarding the tubular activation pathway in IgAN, urinary properdin levels were also significantly increased compared with healthy controls and associated with proteinuria, proximal tubular injury markers and tubulointerstitial fibrotic lesions [24, 55]. In biopsies, properdin was found to be deposited not only in the mesangium, but also at the apical side of the tubules [55]. In the same study, while urinary mannose-binding lectin (MBL) and urinary C4d levels were associated with proteinuria and tubulointerstitial fibrotic lesions, the deposition of MBL and C4d was only found at the mesangium and not at the apical side of tubular cells [55]. Other studies have shown that C4d is deposited in the tubular epithelium under proteinuric conditions. However, the deposition did not correlate with tubular inflammation or fibrosis [116, 117]. Together, these data suggest that the AP might be the main initial activation pathway in the tubular complement activation in IgAN and contributes to disease progression.

### Primary membranous nephropathy (PMN)

PMN is a kidney-specific, podocyte-specific, autoimmune glomerular disease and one of the most common causes of nephrotic syndrome in adults [118]. Patients with PMN have a significant increase in urinary sC5b-9 levels, whereas the plasma sC5b-9 levels are not elevated [27, 51, 52, 56, 106, 119, 120]. In addition to C5b-9 deposition in the glomerulus, immunohistochemical evaluation in some PMN patients showed C5b-9 deposition at the apical side of the tubules [52], suggesting tubular complement activation.

In addition to urinary sC5b-9 levels, urinary Bb and properdin levels were significantly higher compared with healthy controls and significantly correlated with the degree of proteinuria [56]. In a more recent study, urinary Ba, a fragment from factor B upon cleavage, is significantly correlated with urinary sC5b-9 [120]. To further confirm the activation pathway in PMN, a biopsy evaluation is needed. An early study showed that C3 without C1q or C4 is deposited in the tubules of these patients [92]. In a more recent study, biopsy evaluation from three PMN patients also showed that properdin is found at the apical side of the proximal tubules [94]. Together, available evidence indicates that the complement system is activated in the tubular area via the AP.

Several animal models have been developed for PMN, with the passive Heymann's nephritis (PHN) rat model being the most widely used model to study this disease [121]. However, contrary to observational human studies, studies using the PHN rat model showed conflicting results, where some investigators discovered C5b-9 deposition in the proximal tubules [122, 123] and others did not [124, 125]. It is speculated that the tubular brush border in the PHN rat model is more readily sloughed to proteinuria-induced injury or complement activation than in humans [52]. Therefore, deductions from the PHN rat model give limited insights into understanding the mechanism and role of tubular complement activation in human PMN.

### Lupus nephritis (LN)

LN is one of the most worrisome organ-specific manifestations of systemic lupus erythematosus (SLE). Proteinuria is the most common manifestation of LN and approximately half of patients are in the nephrotic range [126]. The plasma sC5b-9 level of patients with LN was significantly higher than that of healthy individuals and significantly higher than that of SLE patients without LN [127–129]. In the kidney, glomerular C5b-9 deposition is a common finding [20, 129, 130]. Next to the elevated plasma level of sC5b-9, the urinary sC5b-9 level is also significantly elevated in patients with LN [58, 106, 128, 129]. While there is sufficient evidence demonstrating intratubular complement activation in patients with LN, there are insufficient data on intratubular complement activation from biopsy findings. As recently reviewed by Koopman *et al*. [20], there are a number of studies that evaluate the tubular deposition of C5b-9 in the biopsies of patients with LN. However, those studies only mentioned the deposition of C5b-9 in the tubular basement membrane. The authors did not explicitly state whether or not there is C5b-9 deposition at the apical side of the tubules.

Concerning the tubular complement activation pathway, Ganguly *et al*. [131] showed that the increase in urinary C3d is not accompanied by an increase in urinary C4d. Another study showed that urinary Ba and C5a correlate with tubular atrophy and interstitial fibrosis, but it does not correspond with the plasma level [128]. Recently it has been reported that urinary C3 and factor I, but not other components from LP or CP, are associated with a higher degree of tubular atrophy and interstitial fibrosis [132]. Together, the current evidence suggests that the complement activation pathway is via the AP.

### C3 glomerulopathy (C3G)

C3G is a group of kidney diseases that is caused by uncontrolled complement activation, characterized by deposition of C3 in the glomerulus in the absence or near absence of immunoglobulin deposition [133]. Defective control due to genetic mutations or acquired antibodies in the complement components in the AP has been discovered as the main cause of complement activation in this disease [134, 135].

Some patients with C3G had elevated urinary sC5b-9 levels [51, 119], suggesting that tubular complement activation occurs. Although kidney biopsies are needed to establish the diagnosis of C3G, and tubular atrophy and interstitial fibrosis can be found in more than half of patients with C3G [136–141], to date, no study has been done to evaluate the presence of tubular complement deposition, including its activation pathway in the biopsy of patients with C3G. Thus future studies evaluating complement deposition in kidney biopsies of patients with C3G and whether its presence is associated with the degree of tubulointerstitial injury are still needed.

### Diabetic nephropathy (DN)

DN is one of the most common complications of diabetes, and it is the leading cause of end-stage kidney disease worldwide. Clinically it is characterized by a gradual increase in proteinuria and a progressive decrease in kidney function [142]. While the systemic complement system is not increased or correlated with proteinuria or estimated glomerular filtration rate (eGFR) in patients with DN [27, 59, 101, 143], the amount of deposited C5b-9 in the glomerulus was significantly higher compared with healthy controls, and it also correlated with the severity of DN [20, 143–145]. Of the three complement activation pathways, current evidence indicates that the complement system is activated via the LP [146].

In contrast to the plasma level, urinary sC5b-9 significantly increased in patients with DN compared with healthy controls, and it correlates with proteinuria and eGFR [27, 59, 101, 147, 148]. There are data on the presence of sC5b-9 deposition at the apical side of the tubules in patients with DN. One study found that DN patients with elevated urinary sC5b-9 levels had heavy deposits of sC5b-9 at the apical side of the tubules despite limited C5b-9 deposition in the glomeruli [52]. This showed that there is local complement activation in the tubules, independent of activation in the glomerulus.

Sun *et al*. [149] showed that factor B^−/−^ diabetic mice had significantly lower tubulointerstitial injury, pointing towards a role for the AP. In line with this, observational studies have shown that the level of urinary factor B significantly increased in patients with DN compared with healthy controls [101, 148]. This indicates that tubular complement activation might occur via the AP in patients with DN.

## ANTI-COMPLEMENT THERAPIES

To date, there are seven complement-inhibiting drugs that have been approved for use in clinical practice: eculizumab (anti-C5 antibody), ravulizumab (anti-C5 antibody), avacopan (C5a receptor antagonist), pegcetalopan (C3 inhibitor), sutimlimab (anti-C1s antibody), danicopan (factor D inhibitor) and iptacopan (factor B inhibitor) [21, 150, 151]. Both eculizumab and ravulizumab have been reported to reduce the amount of sC5b-9 in the urine [152, 153]. However, since the C5b-9 membrane attack complex plays a vital role in eliminating pathogens [15], the incidence of infection, particularly meningococcal infection, was reported to be significantly increased in patients taking eculizumab [154, 155]. Therefore, targeting the specific activation pathway related to disease pathogenesis might be a better approach, as it leaves the immune function of the other pathways intact.

As discussed above, complement activation at the apical side of the tubules in proteinuria is most likely to be activated via the AP. Both drugs that inhibit AP complement activation (danicopan and iptacopan) were approved for the treatment of patients with paroxysmal nocturnal haemoglobinuria (PNH) [150, 151]. However, danicopan failed to adequately inhibit AP complement activation in patients with C3G [156]. In contrast, iptacopan showed promising results. In the phase 2 clinical trial for C3G, iptacopan markedly reduced urinary sC5b-9 levels [157]. Findings from another phase 2 clinical trial for IgAN report similar results [158]. Another factor D inhibitor, vermircopan, is currently being investigated in clinical trials for LN and IgAN patients [21]. Other approaches to inhibit AP complement activation, such as the C3b‐specific nanobody inhibitor EWE‐hC3Nb1, which binds to the active complement component C3b [159], and *Ixodes scapularis* salivary protein Salp20, which binds to properdin [95, 160], are currently being studied at the preclinical level. Expanding the clinical target of AP inhibitors to patients with any proteinuric kidney diseases in combination with anti-proteinuric medication may potentially prevent the progression toward kidney failure. To monitor treatment efficacy, sC5b-9 measurement in the urine (alone or in combination with properdin) may serve as a tool.

## CONCLUSION AND FUTURE PERSPECTIVES

The presence of the terminal complement complex sC5b-9 in proteinuric urine indicates local complement activation at the tubular level. Evidence from clinical and experimental studies strongly suggests that when non-selective proteinuria occurs, the complement system contributes to the tubulointerstitial injury and worsening of kidney function, regardless of the primary kidney disease. The susceptibility of the apical side of proximal tubules for complement activation during proteinuria may be related to the low expression of CRPs in this segment. Despite the lack of studies in some proteinuric kidney diseases, particularly in regard to the histological findings of complement deposition at the apical side of the tubules, currently available evidence has consistently shown that the AP is the main complement activation pathway at the apical tubular level. This is also supported by findings from observational studies, where urinary properdin was strongly associated with urinary sC5b-9 and worse kidney outcomes in patients with various native kidney diseases and kidney transplant recipients [26, 161]. Considering the deleterious effect of tubular complement activation in the progression of kidney injury, the development of therapeutic modalities to inhibit AP activation at the tubular level should be prioritized. This would allow for a better assessment of the role of tubular complement activation in the progression of proteinuric kidney disease and, if relevant, open pathways to new therapies that would potentially benefit all patients with non-selective proteinuria, no matter the underlying cause.

## Data Availability

No new data were generated or analysed in support of this research.
